# Impact of Low-Dose Ketamine Infusion on Intracranial Pressure and Hemodynamics in Septic Shock Patients

**DOI:** 10.1007/s12028-025-02302-4

**Published:** 2025-06-18

**Authors:** Essamedin M. Negm, Hossam Tharwat Ali, Hanaa A. Nofal, Ahmed Mosallem, Ashraf Elsayed Ahmed, Ahmed Ali Morsy, Tamer S. Elserafy, Marwan Elgohary, Khaled Mohamed Altaher, Sherif Sharaf EL Deen, Hani A. Albialy, Ahmed M. Gouda, Ahmed Beniamen

**Affiliations:** 1 Department of Anesthesia, Intensive Care, and Pain Management, Faculty of Medicine, Zagazig University Hospital, Zagazig, Egypt; 2 Qena Faculty of Medicine, South Valley University Hospitals, Qena, Egypt; 3 Community, Environmental Occupational Medicine Department, Faculty of Medicine, Zagazig University, Zagazig, Egypt; 4 Department of Neurosurgery, Faculty of Medicine, Zagazig University Hospital, Zagazig, Egypt; 5 Department of Neurology, Faculty of Medicine, Zagazig University Hospital, Zagazig, Egypt; 6 Department of Internal Medicine, Faculty of Medicine, Zagazig University Hospital, Zagazig, Egypt; 7 Department of Radiodiagnosis, Faculty of Medicine, Zagazig University Hospital, Zagazig, Egypt; 8 Department of Ophthalmology, Faculty of Medicine, Zagazig University Hospital, Zagazig, Egypt; 9 Department of Pharmacy Practice, Faculty of Pharmacy, Zagazig University, Zagazig, Egypt

**Keywords:** Septic shock, Intracranial pressure, Ketamine, Transcranial Doppler, Optic nerve sheath diameter

## Abstract

**Background:**

Septic shock is a recognized cause of global mortality in intensive care units. Sedation and analgesia management are essential for patients with sepsis or hemodynamic instability. Although considered safe concerning hemodynamic changes, ketamine use might cause a substantial rise in intracranial pressure (ICP).

**Methods:**

An interventional study was conducted at the intensive care unit of Zagazig University Hospitals from December 2021 to March 2023 and covered 100 adult patients with septic shock requiring mechanical ventilation, sedation, and vasopressors. Patients with acute brain injury were excluded. Noninvasive ICP including ICP derived from pulsatility index, ICP derived from diastolic flow velocity (ICP_FVd_), and ICP derived from optic nerve sheath diameter, and hemodynamic monitoring were measured before adjunct low-dosage (0.3 mg/kg/hr) continuous ketamine infusion (T0), after 12 h (T1), and after 24 h of infusion (T2).

**Results:**

Baseline ICP derived from optic nerve sheath diameter, ICP derived from pulsatility index, and ICP_FVd_ medians were 14.5 (interquartile range [IQR] 7), 16.8 (IQR 0.91), and 13.8 (IQR 9.38) mm Hg, respectively. Only ICP_FVd_ showed a significant slight increase from 13.75 (IQR 8.5) at T1 to 13.90 (IQR 8.5) at T2 (*P* value = 0.042). The baseline median noninvasive cerebral perfusion pressure was 74.56 (IQR 12.39) mm Hg without significant change at T1 or T2 (*P* value = 0.09). The respiratory rate, heart rate, and mean arterial blood pressure showed no significant changes across timepoints (*P* values = 0.95, 0.86, and 0.14, respectively). The median doses of midazolam, fentanyl, and norepinephrine significantly decreased across the study timepoints, especially at the first 12 h (*P* value < 0.001 for each).

**Conclusions:**

The present pilot study showed promising results of low-dose continuous ketamine infusion adjunctly on ICP and hemodynamics with a substantial reduction of sedatives and vasopressor dose. Further studies with large sample sizes and longer duration of administration and follow-up are needed to expand the current findings.

**Electronic supplementary material:**

The online version of this article (10.1007/s12028-025-02302-4) contains supplementary material, which is available to authorized users.

## Background

Severe sepsis represents a significant challenge in health care, with an incidence reported at 1–2% of all hospital admissions and a ranking as the second leading cause of mortality among patients in intensive care units (ICUs) [1]. Despite advancements in critical care and enhanced insights into the underlying mechanisms of sepsis over the past decade, mortality rates remain elevated, ranging between 30 and 50% [2].

Sepsis-associated encephalopathy often presents with disturbances in the sleep–wake cycle, cognitive impairments, delirium, and even coma [3]. Early identification of intracranial hypertension is essential for timely treatment and improved outcomes, as cerebral edema, a common complication of sepsis-associated encephalopathy, contributes significantly to mortality in patients with sepsis [4]. The gold standard for intracranial pressure (ICP) measurement is the intraventricular catheter; however, its invasive nature poses certain limitations [5]. Noninvasive ICP (nICP) assessment has emerged as a promising alternative for both adult and pediatric populations. Techniques such as measuring the optic nerve sheath diameter (ONSD) via ocular ultrasonography offer a safe, rapid, reliable, and reproducible method for ICP evaluation [6]. Similarly, transcranial Doppler ultrasonography (TCD) provides a noninvasive assessment of ICP and cerebral perfusion pressure (CPP) [7]. For patients with sepsis without intracranial infections, invasive ICP monitoring is generally not recommended [4]. In such cases, nICP methods can be valuable for determining whether ketamine infusion may lead to pathologic increases in ICP.

It can be a challenge to manage sedation and analgesia in the ICU, particularly for patients with sepsis and unstable hemodynamics. Opioids and benzodiazepines may worsen tissue perfusion by lowering cardiac contractility, increasing vasodilation, and decreasing respiratory drive, all of which can thereby add to the pathophysiology of shock [8]. Although benzodiazepines were traditionally the main sedatives used in critically ill patients, nonbenzodiazepines such as propofol and dexmedetomidine have gained popularity in recent years [9]. With only small-sample-sized studies investigating cerebral perfusion in sepsis, current guidelines recommend early antibiotic initiation with fluid resuscitation, whereas there are few recommendations for sedation in septic shock requiring vasopressor support [10, 11].

Ketamine, a nonbarbiturate anesthetic, exerts its effects by blocking N-methyl-D-aspartate and glutamate receptors in a noncompetitive manner. Its use in critical care is gaining traction among physicians for sedating critically ill patients [12, 13]. The 2018 Pain, Agitation/Sedation, Delirium, Immobility, and Sleep Disruption guidelines recommend low-dose ketamine as an adjuvant to opioid therapy, aiming to reduce opioid consumption in postsurgical adult ICU patients [9]. Ketamine’s properties make it particularly suitable for patients with respiratory or hemodynamic instability. Additionally, it influences multiple pathways involved in the inflammatory cascade, offering potential therapeutic benefits during sepsis [14].

Early research suggested that ketamine use might temporarily increase ICP [15], particularly in individuals with preexisting intracranial hypertension and cerebrospinal fluid flow obstruction, in which compromised CPP was observed. This finding contributed to the longstanding perception that ketamine is unsuitable for patients with traumatic brain injury. However, subsequent studies have shown that ketamine’s effects on cerebral hemodynamics and ICP are more complex, varying based on factors such as the concurrent use of other anesthetic agents and the patient’s arterial carbon dioxide levels [16–18].

Despite the increasing interest in ketamine use in critical patients, data on its use in shocked patients without primary neurological injury are scarce. The 2018 Pain, Agitation/Sedation, Delirium, Immobility, and Sleep Disruption guidelines recommendation on its use on ICU patients was limited by the inclusion of trials on postabdominal surgery patients only [9]. A recent pilot trial, the ATTAINMENT trial, showed promising results regarding the safety of low-dose ketamine in mechanically ventilated patients without negative impacts on outcomes including hemodynamics [14]. However, their investigation did not include an assessment of ICP, and their inclusion was not solely for patients with septic shock. Therefore, this study aimed to detect the association of septic shock with ICP changes and to assess the impact of low-dose ketamine infusion as a sedative tool for patients with septic shock.

## Patients and Methods

### Study Design and Population

This prospective interventional clinical study was carried out in the medical and surgical ICUs of Zagazig University Hospital, which comprises nine specialized hospitals, with a total capacity of 2,252 beds. The study included four ICUs: the medical ICU (62 beds), surgical ICU (72 beds), pulmonary ICU (15 beds), and coronary care unit (16 beds). Conducted over 15 months from December 2021 to March 2023, the study involved 100 patients with septic shock aged over 18 years who required mechanical ventilation, sedation with midazolam and fentanyl, and vasopressor therapy with norepinephrine (NE), the primary vasopressor used in these ICUs. The applicable parts of the Consolidated Standards of Reporting Trials guidelines were followed to report and write this trial.

Patients were diagnosed based on their clinical presentations and confirmed laboratory and radiological data, following the currently implemented evidence-based protocols [19]. Patients with a history of previous intracranial surgery or associated with any intracranial pathology, any increased or decreased ICP based on clinical history, and neurological conditions or acute brain injury (abnormal neurologic examination performed before the introduction of sedation suggestive of primary neurological condition) were excluded. Inflammatory lesions of the optic nerve itself (optic neuritis); trauma to the eye such as rupture globe, glaucoma, and optic atrophy; pregnant patients; and patients with end-stage liver, renal disease, a history of dementia or psychiatric disorders, or intake of any antipsychotic or antidepressant medications at home were deemed ineligible. Patients in the perioperative time frame, patients with acute coronary syndrome, those diagnosed with other types of shock, and those with known allergies to ketamine were also excluded from the study.

### Sample Size Calculation

Sample size calculation was based on mean ICP before and after ketamine infusion on noninvasive estimators of intracranial pressure retrieved from previous research [20]. Using G power program version 3.1.9.4 to calculate sample size based on the effect size of 0.355, using a two-tailed test, α error of 0.05, and power of 90%, the total calculated sample size would be 85, and by adding 15% to compensate for possible dropout, then the total sample size would be 100 (Supplementary Fig. S1).

### Data Collection and Measurements

#### Baseline, Laboratory, and Clinical Data

Patient characteristics, clinical data (history, diagnosis, and cause of admission), localization of sepsis source, type and dose of sedation, and type and dose of vasopressor were first registered. Hemodynamic monitoring parameters included invasive blood pressure (BP), heart rate (HR), and respiratory rate (RR). Laboratory investigations included c-reactive protein, procalcitonin lactates, and kidney and liver functions. Ventilation and oxygenation parameters including airway peak pressure, airway plateau pressure, and end-tidal CO_2_ were also collected. Acute Physiology and Chronic Health Evaluation II score, Sequential Organ Failure Assessment score, Glasco coma scale (GCS), and Full Outline of Unresponsiveness score were also calculated [21, 22].

#### ICP Noninvasive Estimation

Ultrasonographic measurement of ONSD was performed by a single trained investigator with more than 200 ultrasound examinations in the past 3 years. Color Doppler-Ultrasound equipment (Siemens Acuson X300 ultrasound machine; Siemens Medical Solutions USA Inc., Malvern, PA) with a linear 7.5-MHz ultrasound probe was gently placed in the supine positions on the closed upper eyelid without applying pressure to the eye. ONSD measured 3 mm behind the globe in the two-dimensional mode using an electronic caliper [23] (Supplementary Fig. S2). Each optic nerve had two measurements, one in the sagittal plane and the other in the transverse plane. The mean of the four values recorded in each patient to obtain the final ONSD measurement was used. To avoid any potential harm to the ocular structures, we followed the British Medical Ultrasound Society’s guidelines and the as low as reasonably achievable concept [24]. The following estimate method, which is based on the linear regression between the invasive intraparenchymal ICP (in mm Hg) and ONSD (in mm) in a previously reported cohort of neurocritical care patients, was used to determine the value of the ONSD-predicted nICP (nICP_ONSD_ = 5 × ONSD − 14) [7].

TCD measurements were performed by a single investigator with three years of experience performing TCD in a clinical setting. A P 4–2 phased array 2-MHz probe was used (Supplementary Fig. S3). All patients had both middle cerebral arteries insonated at a depth of 50–60 mm through the transtemporal window located over the zygomatic arch in front of the tragus of the ear. After measuring the middle cerebral artery flow velocity (FV) on both sides, the final FV measurements matched the average of the two values on both sides. Middle cerebral artery FVs (systolic FV, diastolic FV [FVd], and mean FV [FVm]) were recorded. The pulsatility index (PI), which assesses the flow resistance, was calculated (PI = [systolic FV – FVd]/FVm), and any value higher than 1.2 is considered high-resistance flow [25]. Resistance index, which is another less frequently used method for flow resistance, was calculated by subtracting end diastolic velocity (EDV) from peak systolic velocity (PSV) and dividing the value by PSV (PSV – end diastolic velocity/PSV), with normal values being below 0.75 [26]. The ICP derived from PI (ICP_PI_) was determined according to a formula based on data given by Cardim et al. [27] (ICP_PI_ = 4.47 × PI + 12.68). Noninvasive CPP (nCPP) derived from FVd has been estimated as follows: (nCPP = ABPm × [FVd/FVm] + 14), according to Czosnyka et al. [28], in which mABP is the mean arterial BP. ICP was then estimated as the difference between the inflow (mABP) and CPP as follows (ICP_FVd_ = mABP – nCPP) which puts into the following equation $\left( {{\mathrm{ICPFVd}}\, = \,{\mathrm{mABP}} \left[ {1 {-} \frac{{{\mathrm{FVd}}}}{{{\mathrm{FVm}}}}} \right]{-} 14} \right)$.

For ICP estimation, ONSD and TCD values were measured and reported before and after ketamine infusion. Noninvasive ICP monitoring was measured before ketamine infusion (T0), after 12 h of ketamine infusion (T1), and after 24 h of ketamine infusion (T2). The readings were taken away from any endotracheal suctioning, patient straining, or patient positioning with normalization of physiological and pathological factors influencing ICP. We kept the average volume status (roughly by central venous pressure and by other static and dynamic volume status monitoring parameters). Ventilator parameters were adjusted in a range not influencing the ICP with the normalization of arterial carbon dioxide level.

#### Study Interventions

The intervention group received a continuous low-dose infusion of ketamine in addition to the standard ICU sedation protocol. Ketamine was administered at a fixed rate of 5 μg/kg/min (0.3 mg/kg/hr) [29] for 24 h. Based on existing literature, ketamine doses can be safely titrated up to 15 μg/kg/min [30, 31] as needed to achieve adequate sedation. Decisions regarding continued ketamine use or other sedatives and analgesics beyond the 24-h trial period were left to the discretion of the treating physicians and were not part of the study’s objectives. Physicians could also supplement sedation with additional medications as necessary to achieve the targeted Richmond Agitation-Sedation Scale level or discontinue ketamine at any time if deemed clinically appropriate. Deep sedation was primarily indicated to facilitate mechanical ventilation and minimize patient-ventilator asynchrony in the context of severe sepsis-induced respiratory failure. As for sedative titrations, fentanyl and midazolam were titrated to achieve a Richmond Agitation-Sedation Scale score of − 2 to − 3. Boluses were administered as needed to maintain this level of sedation. Regarding vasopressor administration, NE was titrated to maintain a mean mABP of 65–75 mm Hg. The titration parameters were adjusted if the patient showed signs of poor perfusion, or excessive hypertension. Patients were ventilated using a lung-protective strategy with tidal volumes of 6–8 mL/kg predicted body weight and positive end-expiratory pressure titrated to optimize oxygenation and minimize plateau pressure. Ventilator parameters were adjusted in a range not influencing the ICP (high positive end-expiratory pressure was excluded) with the normalization of arterial carbon dioxide level. Weaning from mechanical ventilation was initiated when patients met standard criteria, including hemodynamic stability, adequate oxygenation, and spontaneous breathing trials.

#### Outcome Measures and Study End Points

The primary outcomes were baseline ICP in patients with sepsis in addition to the change in ICP after ketamine infusion. The secondary outcomes include hemodynamic changes (BP, HR), vasopressor dose, and other sedative doses.

#### Ethical Approval and Trial Registration

The current study granted the ethical approval of the Faculty of Medicine, Zagazig University (ZU-IRB no. 6928–6-9–2021), and it was registered at clinicaltrials.gov (NCT05149586) and carried out per the revised declaration of Helsinki. Before enrollment, as patients could not decide to participate in this study, written informed consent was obtained from first-degree relatives of all participants.

#### Statistical Analysis and Data Interpretation

Statistical analysis was performed using SPSS software version 25 (SPSS Inc., PASW Statistics for Windows, version 25; SPSS Inc., Chicago, IL). Qualitative data were expressed as frequencies and percentages, whereas quantitative data were summarized as median (interquartile range) for nonnormally distributed variables and mean ± standard deviation (SD) for normally distributed variables. The Kolmogorov–Smirnov test was applied to assess normality. Statistical significance was determined at a *P* value of ≤ 0.05. We implied repeated measure analysis of variance test with post hoc Bonferroni test and Friedman test with post hoc Nemenyi test, for parametric and nonparametric variables respectively, to compare readings of each variable.

## Results

The present study conducted at medical and surgical ICUs covered 100 patients with septic shock who required mechanical ventilation, sedation (midazolam and fentanyl), and vasopressor therapy using NE, without any missing data. The mean age ± SD of the studied patients is 51.12 ± 9.71, ranging from 29 to 67, with 54% being men. Around 27% had diabetes, 17% had hypertension, and 12% had ischemic heart disease as shown in Table 1. The primary causes of ICU admission included septic shock (61%), respiratory failure (16%), life-threatening arrhythmia (6%), and acute coronary syndrome (5%). Sepsis was distributed as follows: pneumonia (50%), urinary tract infection (28%), blood (25%), soft tissue infection (18%), abdominal infection (12%), and surgical site infection (2%), as shown in Table 1.

**Table 1:** Sociodemographic data and medical history of the patients

Variable	Frequency (*N* = 100)	Percentage (%)
Age in years	51.12 ± 9.71 # (29–67) ##	
Sex - Male - Female	54 46	54.0 46.0
Past medical history - Malignancy - Hepatitis C virus - Smoking - Deep venous thrombosis - Ischemic heart disease - Asthmatic/COPD - Thyroid diseases - Diabetes mellitus - Hypertension - Dyslipidemia - Chemotherapy - Steroid therapy - Others ###	11 2 9 1 12 6 2 27 17 7 5 2 4	11.0 2.0 9.0 1.0 12.0 6.0 2.0 27.0 17.0 7.0 5.0 2.0 4.0
The primary cause of ICU admission - Uncontrolled hypertension - Septic shock - Respiratory failure - Pulmonary edema - Pulmonary embolism - Life-threatening arrhythmia - GIT bleeding - Diabetic ketoacidosis - Acute coronary syndrome	1 61 16 3 2 6 2 4 5	1.0 61.0 16.0 3.0 2.0 6.0 2.0 4.0 5.0
Sepsis localization - Mediastinitis/osteomyelitis - Abdominal infection - Surgical site infection - Urinary tract infection - Pneumonia - Bloodstream infection - Soft tissue infection - Negative culture (unknown origin) - Empyema	2 12 2 28 50 25 18 2 2	2.0 12.0 2.0 28.0 50.0 25.0 18.0 2.0 2.0

^#^ mean ± standard deviation

^##^ (Range)

^###^ splenectomy, COVID-19, radiotherapy, and SLE

Abbreviations: COPD: chronic obstructive pulmonary disease; GIT: Gastrointestinal

Table 2 illustrates that the mean GCS was 14, the mean Full Outline of Unresponsiveness score was 12.22, the median Acute Physiology and Chronic Health Evaluation II score was 18 ranging from 11 to 38, and the median Sequential Organ Failure Assessment score was 8 ranging from 2 to 14. As for the laboratory findings, the median procalcitonin was 20 ranging from 0.5 to 100 ng/ml, the median c-reactive protein was 197 ranging from 50 to 345 mg/dL and the mean ± SD lactate was 4.92 ± 1.59. The median WBCS was 15 ranging from 2 to 45 (10^9^/L) and the median platelet count was 167 ranging from 33 to 478 (10^9^/L). The median creatinine was 1.6 ranging from 0.7 to 2.3 mg/dL, median aspartate transaminase was 52 ranging from 20 to 200 U/L, median alanine transaminase was 52 ranging from 20 to 200 U/L, median alanine transaminase was 35 ranging from 22 to 177 U/L. The details of the laboratory baseline findings of the patients are shown in Table 2.

**Table 2:** Clinical and laboratory findings of the study patients

Variable	Value (*N* = 100) #
GCS	14.06 ± 0.96
FOUR score	12.22 ± 1.16
APACHE score	18 (9)
SOFA score	8.0 (3)
PCT (ng/ml)	20 (40)
CRP (mg/dL)	197 (95)
Lactate (mmol/L)	4.92 ± 1.59
WBCs (10^9^/L)	15 (11)
Creatinine (mg/dL)	1.6 (0.9)
AST (U/L)	52 (17)
ALT (U/L)	35 (17)
Albumin (g/dL)	2.9 (1.1)
Platelet (10^9^/L)	167 (123)
Total bilirubin (mg/dL)	1.66 (2.21)
Direct bilirubin (mg/dL)	1.04 (1.8)

^**#**^ Values are provided in mean ± standard deviation or median (interquartile range)

Abbreviations: GCS: Glasgow coma scale; FOUR: Full Outline of Unresponsiveness; APACHE: The Acute Physiology and Chronic Health Evaluation; SOFA: Sequential Organ Failure Assessment; PCT: Procalcitonin; CRP: C-reactive protein; WBC: White blood cell; AST: Aspartate transaminase; ALT: alanine transaminase

Baseline (T0) ICP_ONSD_, ICP_PI_, and ICP_FVd_ medians were 14.5 (interquartile range [IQR] 7), 16.8 (IQR 0.91), and 13.8 (IQR 9.38) mm Hg, respectively. Baseline resistance indexes and PIs were 0.577 ± 0.07 and 0.92 (0.29), respectively. The baseline median nCPP was 74.56 (IQR 12.39) mm Hg and increased to 75.35 (IQR 12.22) and 75.0 (IQR 12.09) mm Hg at T1 and T2 respectively with no significant differences (*P* value = 0.09). Out of ICP measures, only ICP_FVd_ showed a significant slight increase from 13.75 (8.5) at T1 to 13.90 (8.5) at T2 (*P* value = 0.042) without a significant difference between T0 and T1 (*P* value = 0.8) or T0 and T2 (*P* value = 0.94). The baseline RR was 24 (8) bpm, whereas the baseline HR was 90 (14) bpm. The RR, HR, and mABP showed no significant changes across timepoints (*P* values = 0.95, 0.86, and 0.14 respectively). The details of ICP and hemodynamic measures at different timepoints are shown in Table 3.

**Table 3:** ICP and hemodynamic measurements at study time points

Variable	T0 #	T1 #	T2 #	*P value #*	Post hoc *(P value) ##*
RR in bpm	24.0 (8)	24.0 (8)	24.0 (8)	0.95	-
HR in bpm	90.0 (14)	90.0 (15)	88.5 (16)	0.86	-
mABP in mmHg	88.0 (11)	88.0 (12)	88.0 (11.92)	0.14	-
Temperature in celsius degrees	37.6 (0.7)	37.6 (0.7)	37.5(0.7)	0.68	-
CVP in cm H_2_O	9.0 (3)	9.0 (2)	9.0 (3)	0.71	-
ETCo2 in mmHg	34.5 (6)	34.0 (6)	35.0 (6)	0.96	-
P/F ratio	198.0 (100)	197.5 (100)	198 (100)	0.96	-
Peak pressure in cm H_2_O	24.5 (3)	24.0 (3)	24.0 (3)	0.49	-
Plateau pressure in cm H_2_O	21.0 (3)	21.0 (3)	21.0 (3)	0.83	-
ONSD in mm	5.7 (1.4)	5.7 (1.4)	5.7 (1.5)	0.98	-
ICP_ONSD_	14.5 (7)	14.5 (7)	14.5 (7.5)	0.92	-
RI	0.577 ± 0.07	0.576 ± 0.07	0.576 ± 0.072	0.37	-
PSV in cm/s	86.85 ± 26.08	86.92 ± 25.58	86.98 ± 25.86	0.68	-
EDV in cm/s	34.85 (21.2)	34.9 (20.7)	34.85 (20.4)	0.27	-
FVm in cm/s	53.70 ± 17.58	53.66 ± 17.60	53.69 ± 17.57	0.85	-
PI	0.92 (0.29)	0.92 (0.29)	0.92 (0.29)	0.49	-
ICP_PI_ in mmHg	16.8 (0.91)	16.7 (1.03)	16.7 (1.04)	0.12	-
nCPP in mmHg	74.56 (12.39)	75.35 (12.22)	75.0 (12.09)	0.09	-
ICP_FVd_ in mmHg	13.81 (9.38)	13.75 (8.5)	13.90 (8.5)	0.01 *	P1 = 0.804 P2 = 0.94 P3 = 0.042*

^#^ Values are provided in mean ± standard deviation or median (interquartile range) and hence repeated measure ANOVA and Friedman test were used for significance testing respectively

^##^ Post hoc Nemenyi test; P1: difference between T0 & T1, P2: difference between T0 & T2, P3: difference between T1 & T2

Abbreviations: RR: Respiratory rate; HR: Heart rate; mABP: Mean arterial blood pressure; CVP: Central venous pressure; ETCo2: End-tidal Co2; P/F ratio: PaO2/FiO2; ONSD: Optic nerve sheath diameter; ICP_ONSD_: ONSD-derived intracranial pressure; RI: Resistance index; PSV: Peak systolic velocity; EDV: End diastolic velocity; FVm: Mean flow velocity; PI: Pulsatility index; ICP_PI_: PI-derived intracranial pressure; nCPP: Noninvasive cerebral perfusion pressure; ICP_FVd_: FVd-derived intracranial pressure

^*^ Statistically significant

The median dosage of midazolam at baseline was 0.09 (0.06) mg/kg/hr, which decreased to 0.07 (0.06) at T1 and 0.07 (0.05) at T2 (*P* values < 0.001, for both compared with baseline). Similarly, fentanyl dosage was 0.88 (0.23), 0.80 (0.20), and 0.8 (0.20) μg/kg/hr at T0, T1, and T2 respectively (*P* values = 0.04 and < 0.001 for T1 and T2 compared with T0, respectively). Regarding the vasopressor, NE, the baseline dosage was 0.17 (0.16) μg/kg/min, which decreased to 0.15 (0.12) at T1 (*P* value < 0.001) and 0.135 (0.12) at T2 (*P* value < 0.001) (Table 4).

**Table 4:** Doses of sedatives and vasopressor at study time points

Variable	T0 #	T1 #	T2 #	*P value #*	Post hoc *(P value) ##*
Midazolam dosage (mg/kg/h)	0.09 (0.06)	0.07 (0.06)	0.07 (0.05)	< 0.001*	P1 < 0.001* P2 < 0.001* P3 = 0.47
Fentanyl dosage (μg/kg/hour)	0.88 (0.23)	0.80 (0.20)	0.8 (0.20)	< 0.001*	P1 = 0.04* P2 < 0.001* P3 = 0.73
Norepinephrine dosage (μg/kg/minute)	0.17 (0.16)	0.15 (0.12)	0.135 (0.12)	< 0.001*	P1 < 0.001* P2 < 0.001* P3 = 1.0

^#^ Values are provided in median (interquartile range) and hence Friedman test was used for significance testing

^##^ Post hoc Nemenyi test; P1: difference between T0 & T1, P2: difference between T0 & T2, P3: difference between T1 & T2

^*^ Statistically significant

## Discussion

Sepsis causes several systemic changes that can affect the brain, and sepsis-associated delirium may manifest before other organ failure. Data on cerebral perfusion in sepsis are not decisive with most reports including small sample sizes [10]. Current guidelines still place a strong emphasis on management through early capture and treatment with early antibiotics and supportive fluids, whereas there are few recommendations for sedation in septic shock requiring vasopressor support [11]. The current study is the first to explore the impact of adjunct low-dose ketamine infusion on ICP monitoring in critically ill septic shocked and mechanically ventilated patients.

The brain experiences significant changes because of the inflammatory response associated with sepsis. Increased blood–brain barrier permeability may result in significant changes in the regulation of cerebral blood flow and perfusion. Within the context of septic shock, mABP is notably low and CPP is consequently low. Additionally, depending on the degree of blood–brain barrier dysfunction, the effects of vasopressors on the brain may be unexpected [10]. In magnetic resonance imaging research, sepsis-induced lesions in white matter could be seen in some patients with septic shock and neurological dysfunction, suggesting a breakdown in the blood–brain barrier. However, evidence of cerebral edema cannot be always defined [32]. Salahuddin et al. [33] observed that coma in individuals with nontraumatic radiological cerebral edema often presents due to septic encephalopathy.

An adult resting supine usually has an ICP of 7 to 15 mm Hg [34]. In sepsis, mABP levels are often low; therefore, even slight increases in ICP may adversely affect CPP. Patients with sepsis appear to experience moderate ICP rises more frequently, regardless of the amount of fluid given [35]. However, in the current study, baseline ICP_ONSD_ and ICP_FVd_ were below 15 mm Hg, whereas only ICP_PI_ had a median value of 16.8 mm Hg with a baseline median value of PI as 0.92. It is noteworthy that the normal range for PI is usually between 0.6 and 1.2 [25]. In contrast to our results, Pfister et al. [35] assessed ICP noninvasively on 16 patients with sepsis and found that 47% of patients had mild elevations of ICP > 15 mm Hg, an increase > 20 mm Hg was not seen, and 20% of their patients had CPP below 50 mm Hg.

PI-derived methods of ICP measurement (ICP_PI_) are based on the positive correlation between ICP and PI especially during an increased ICP. However, an increase in PI is not always specific to an increase in ICP, such as in cases with decreased arterial BP, that leads to a decrease in CPP without an increase in ICP [36]. In our study, baseline nCPP was 74.56 mm Hg, which lies in the normal range [10]. ICP_FVd_ has been considered a better estimator for ICP than ICP_PI_ [27]. Although the median baseline ICP_ONSD_ was normal, the median ONSD was 5.7 mm. A cutoff point of 5 mm has been usually considered to correlate with an ICP of 20 mm Hg, however, recent observations showed variable ranges according to age, sex, ethnicities, comorbidities, and the operator that can extend the cutoff point as high as 5.7 mm [37, 38].

In our study, baseline nCPP was 74.56 mm Hg, which lies in the normal range with a median mABP of 88 mm Hg [10]. However, cerebral blood flow decreases in patients with sepsis can occur independently of changes in BP or cardiac output [39]. In patients with sepsis who may develop brain edema and an elevated ICP exceeding 15 mm Hg, CPP could fall below 50 mm Hg if mABP is maintained between 60 and 70 mm Hg, as is typically recommended. This might lead to brain hypoperfusion [35]. The concurrent use of sedatives may be a contributing factor to reduced brain perfusion in patients with sepsis [40]. Although our baseline GCS score had a median of 14, some reports revealed lower cerebral blood flow with sepsis-associated delirium than awake controls [39].

For patients with sepsis, sedation, and analgesia in the ICU may be challenging. BP can be lowered and respiratory drive reduced by benzodiazepines and opioids [8, 41]. The routine use of ketamine infusion for primary sedation in the medical ICU adult population with sepsis has not yet been documented in any published reports [42]. Ketamine’s advantages in the ICU environment have only been shown in a limited number of case reports [43, 44]. It appears reasonable to investigate the potential role of ketamine in critical illness. According to the results of this study, no significant change was detected in ICP_ONSD_, ICP_PI_, and ICP_FVd_ at 12 and 24 h after ketamine infusion except for the slight increase in ICP_FVd_ from 13.75 at 12 h to 13.90 at 24 h, which followed a slight decrease from 13.81 at baseline to 13.75 at 12 h, without overall clinically significant change between 24 or 12 h and baseline readings. Several studies examined the impact of ketamine on ICP in adult mechanically ventilated patients admitted to the ICU with traumatic brain injury or subarachnoid hemorrhage; neither pathological ICP nor differences between patients who received ketamine, and the control group were detected [45, 46]. One research found that following a ketamine bolus, ICP measurements decreased rapidly and then rose gradually over time without any sign of risk [47]. Another study reported no overall relationship between ketamine dosage and ICP, although there were tendencies toward lowering ICP at higher ketamine doses [48]. In addition, the systematic review published by Zeiler et al. [46] stated that ketamine does not raise ICP in nontraumatic neurological diseases when patients are ventilated and sedated, and it may even lower it in selected cases.

Our results showed a nonsignificant increase in nCPP following ketamine administration without significant change in hemodynamics. CPP monitoring was included in previous studies [45, 49, 50]. After a bolus of ketamine, there was a reported rise in CPP of 3.9 mm Hg for 2 min without any adverse effects [50]. An increase in CPP and a corresponding decrease in the doses of vasopressors needed to counteract the effect of opioid-based sedatives were also reported [51]. In our cohort, the median dose of NE decreased during the 24 h ketamine infusion with the significant decrease being mainly in the first 12 h. Theoretically, ketamine could be beneficial for patients with hemodynamic instability with insufficient data for solid conclusions. The present findings extend such a hypothesis as no changes were observed in hemodynamic parameters. When compared with propofol or dexmedetomidine for ICU sedation, ketamine was found to have less clinically significant hypotension or bradycardia as well as a lower absolute fall in hemodynamic parameters during the time of sedation [52]. Another study found that ketamine’s cardiovascular stimulatory effects in patients with sepsis with cardiovascular instability lower inotropic support and had a beneficial anti-inflammatory effect against the sepsis process [53]. The median dose of midazolam and fentanyl decreased with the ketamine use especially during the first 12 h, in concordance with some studies that showed an opioid-sparing effect, mostly for ICU patients hospitalized for trauma and postoperative reasons [54, 55]. Minimizing sedation is generally preferred, and low-dose ketamine may offer a potential strategy to reduce the overall sedative burden in this patient population. The present results can pave the way to investigate the possibility of complete replacement of benzodiazepines with ketamine given their high risk for associated side effects.

Although the first to explore the impact of adjunct low-dose ketamine infusion on ICP and hemodynamic monitoring in the context of sepsis with a complete data set, this study has some limitations including short administration and follow-up period of only 24 h. We only evaluated the effect of low-dosage ketamine (0.3 mg/kg/hr), and the impact of higher ketamine doses on ICP remains unclear. Future studies should investigate the dose-dependent effects of ketamine on cerebral hemodynamics in septic shock and the possibility of complete replacement of benzodiazepines with ketamine. Noninvasive ICP monitoring through ONSD ultrasound or TCD is valid, but not the gold standard, and cutoff points can vary although we used a single experienced operator to minimize interoperator variability. Another study limitation was the baseline measurement of the ICP parameters and the CPP while the patient was being resuscitated with fluids and on vasopressors. One main limitation is lacking a control group to detect the effect solely of ketamine, in addition to the small sample size and single-center design, which hinder the generalizability of the results. Long-term, large sample-sized, controlled, randomized, and anonymized studies are recommended to validate our results. We also recommend future studies to include invasive methods in further research on ketamine use to validate the present results generated by noninvasive methods. Future studies should also consider population subgrouping (for example based on relevant assessments) to identify the best candidate to benefit from ketamine infusion while recognizing those with higher risks. For instance, the present study excluded patients with abnormal neurological examination suggestive of neurological conditions that might affect the response of the nervous system to ketamine infusion, and hence future studies can consider such a point to detect changes in that specific population.

## Conclusions

Adult patients with septic shock, on hemodynamic support, had high-to-normal ICP. Low-dose continuous ketamine infusion as an adjunct sedative appeared to have favorable outcomes concerning ICP and hemodynamic monitoring and was associated with a significant reduction in doses of sedative and vasopressor. Further studies with a larger number of patients and longer duration are needed to support the study findings, especially in patients with septic encephalopathy and/or low GCS scores.

## Electronic supplementary material

Below is the link to the electronic supplementary material.

Supplementary file 1 (pdf 19 kb)

## Declarations

### Clinical Trial Registration

ClinicalTrials.gov: NCT05149586.
