# Correction to: Impact of Low-Dose Ketamine Infusion on Intracranial Pressure and Hemodynamics in Septic Shock Patients

**DOI:** 10.1007/s12028-025-02373-3

**Published:** 2025-09-22

**Authors:** Essamedin M. Negm, Hossam Tharwat Ali, Hanaa A. Nofal, Ahmed Mosallem, Ashraf Elsayed Ahmed, Ahmed Ali Morsy, Tamer S. Elserafy, Marwan Elgohary, Khaled Mohamed Altaher, Sherif Sharaf E. L. Deen, Hani A. Albialy, Ahmed M. Gouda, Ahmed Beniamen

**Affiliations:** 1 Department of Anesthesia, Intensive Care, and Pain Management, Faculty of Medicine, Zagazig University Hospital, Zagazig, Egypt; 2 Qena Faculty of Medicine, South Valley University Hospitals, Qena, Egypt; 3 Community, Environmental Occupational Medicine Department, Faculty of Medicine, Zagazig University, Zagazig, Egypt; 4 Department of Neurosurgery, Faculty of Medicine, Zagazig University Hospital, Zagazig, Egypt; 5 Department of Neurology, Faculty of Medicine, Zagazig University Hospital, Zagazig, Egypt; 6 Department of Internal Medicine, Faculty of Medicine, Zagazig University Hospital, Zagazig, Egypt; 7 Department of Radiodiagnosis, Faculty of Medicine, Zagazig University Hospital, Zagazig, Egypt; 8 Department of Ophthalmology, Faculty of Medicine, Zagazig University Hospital, Zagazig, Egypt; 9 Department of Pharmacy Practice, Faculty of Pharmacy, Zagazig University, Zagazig, Egypt

**Correction to** **:** **Neurocrit Care** 10.1007/s12028-025-02302-4

The original article has been corrected to change the dosing of ketamine infusion, which was written as **(0.3 μg/kg/hr)** in the abstract (methods paragraph) and discussion (last paragraph). However, the corrected dose in both abstract and discussion is: **(0.3 mg/kg/hr).**

