# Supplementary material for: Impact of Low-Dose Ketamine Infusion on Intracranial Pressure and Hemodynamics in Septic Shock Patients
Source: Neurocrit Care. 2025 Jun 18;44(1):136–45. doi: 10.1007/s12028-025-02302-4 (PMC12819488; doi:10.1007/s12028-025-02302-4)

Impact of Low-Dose Ketamine Infusion on Intracranial Pressure and Hemodynamics in Septic Shock Patients

Supplementary Data

Figure S1: Graph for sample size calculation by G power program version 3.1.9.4

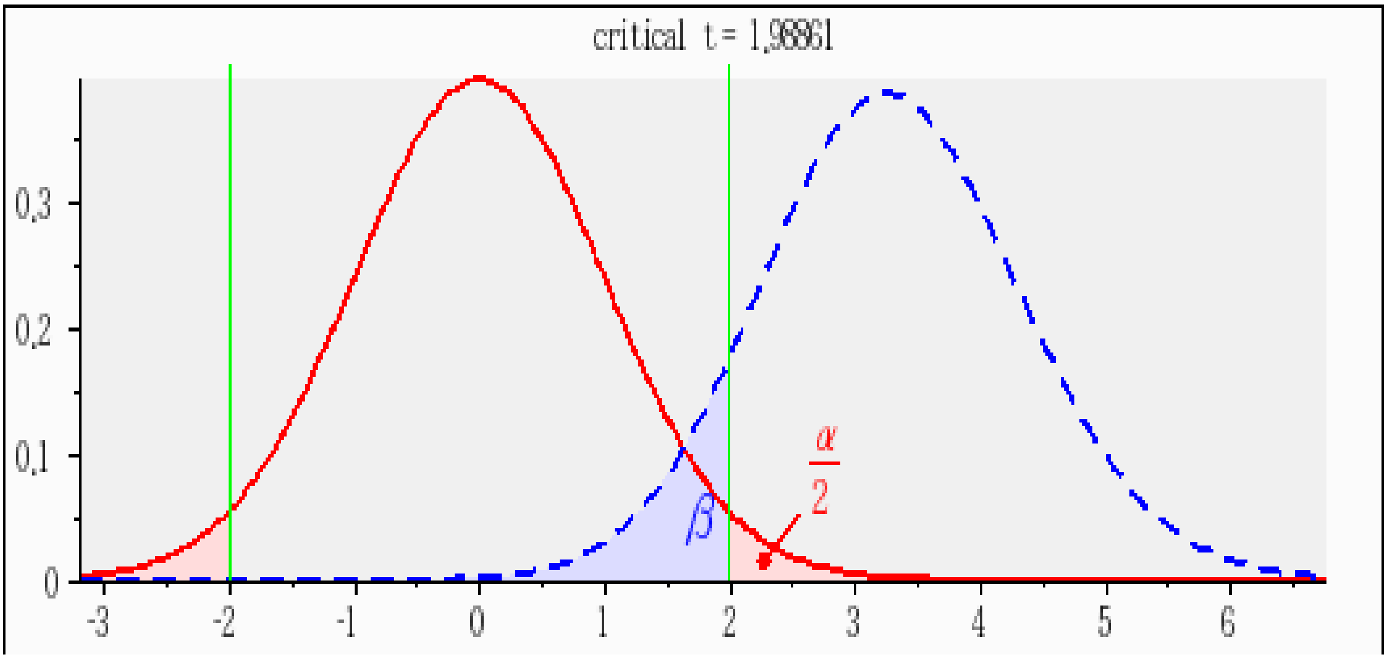

Figure S2: Optic nerve sheath diameter by ultrasonography for septic shock patient

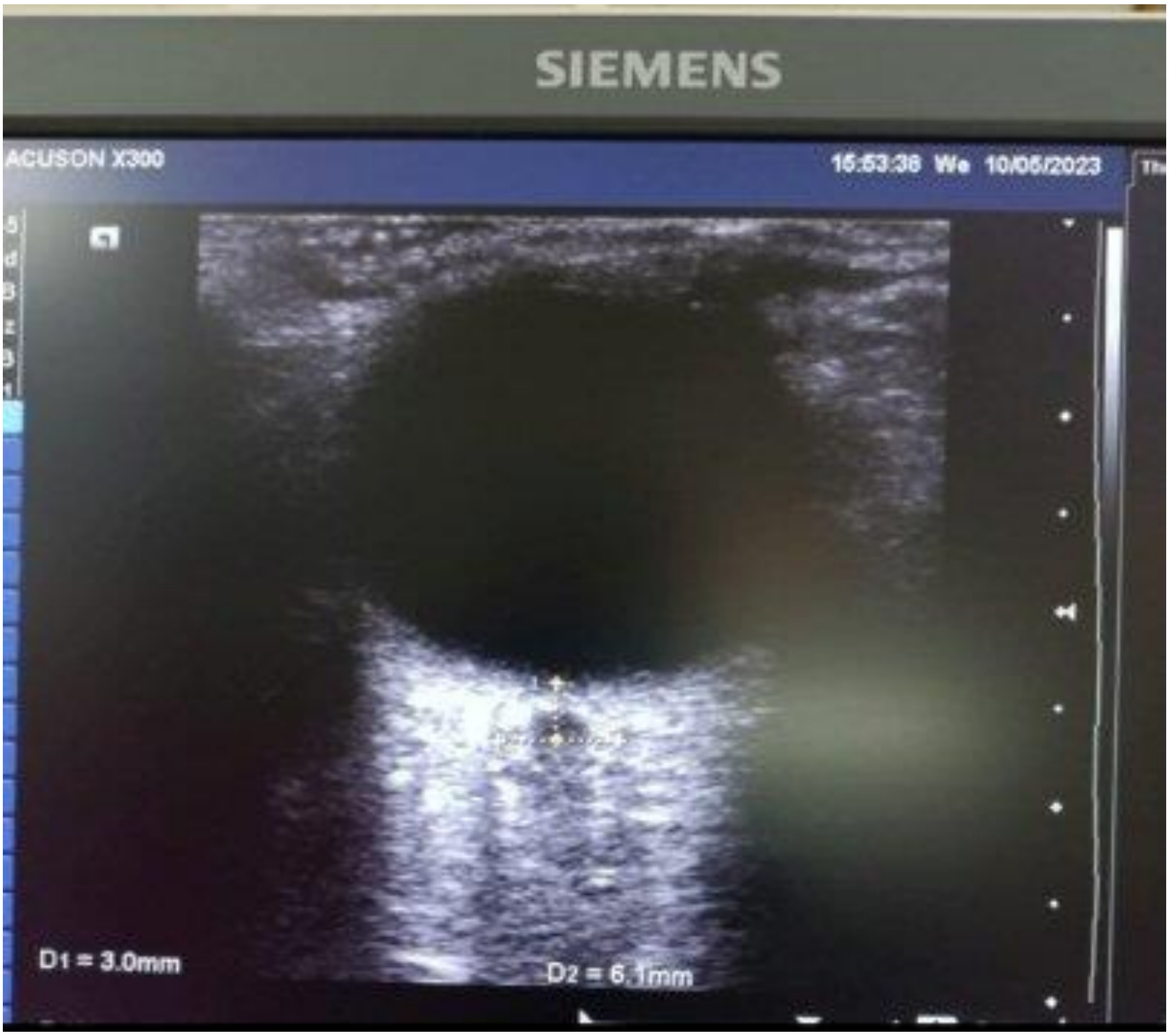

Figure S3: Transcranial Doppler for middle cerebral artery by ultrasonography for septic shock patient

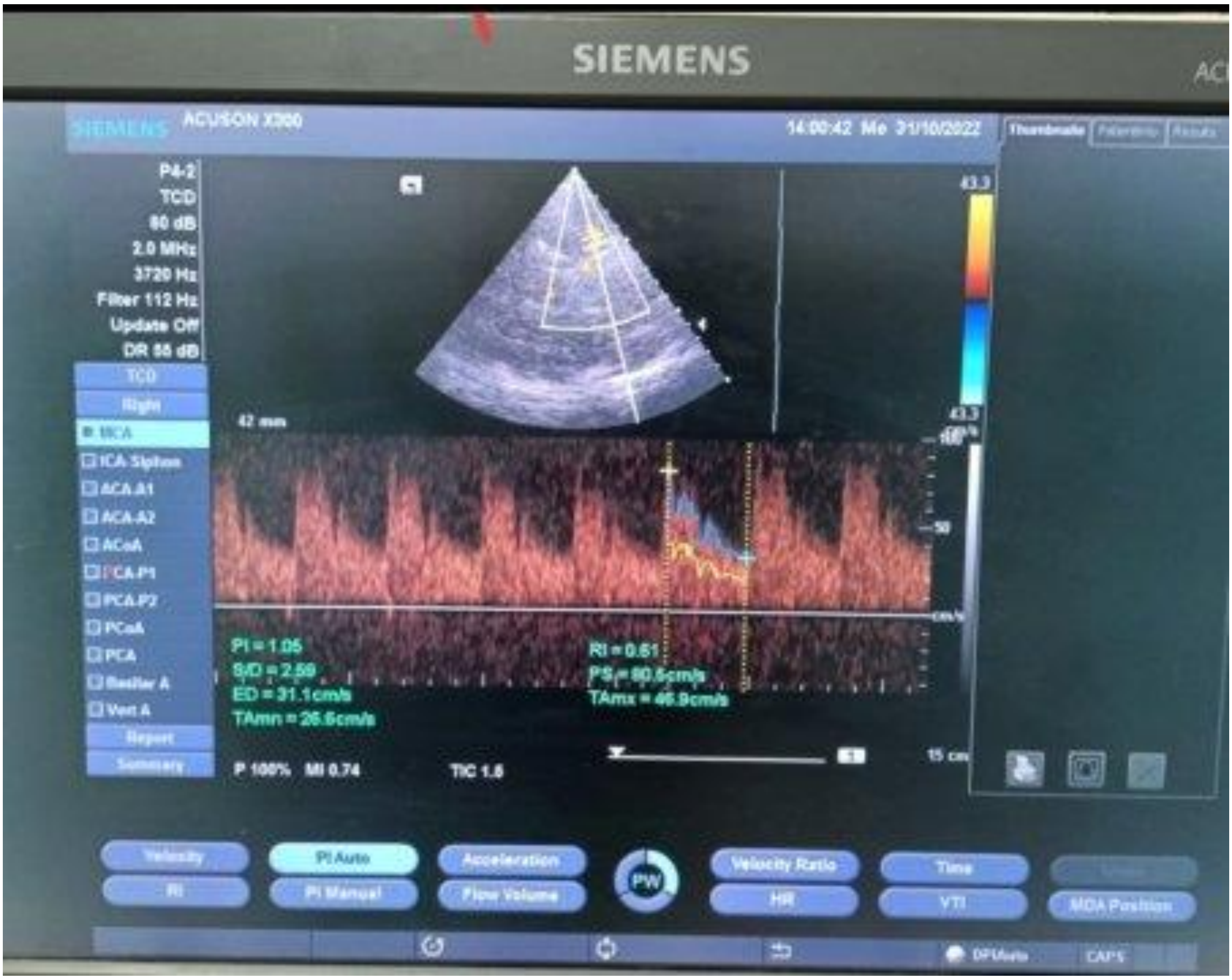

Supplement: Supplementary file 1 — (pdf 19 kb) [file 12028_2025_2302_MOESM1_ESM.pdf]
